# Parameterization of clear-sky surface irradiance and its implications for estimation of aerosol direct radiative effect and aerosol optical depth

**DOI:** 10.1038/srep14376

**Published:** 2015-09-23

**Authors:** Xiangao Xia

**Affiliations:** 1 LAGEO, Institute of Atmospheric Physics, Chinese Academy of Sciences, Beijing, China

## Abstract

Aerosols impact clear-sky surface irradiance (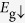) through the effects of scattering and absorption. Linear or nonlinear relationships between aerosol optical depth (*τ*_a_) and 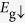 have been established to describe the aerosol direct radiative effect on 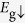 (ADRE). However, considerable uncertainties remain associated with ADRE due to the incorrect estimation of 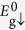 (*τ*_a_ in the absence of aerosols). Based on data from the Aerosol Robotic Network, the effects of *τ*_a_, water vapor content (*w*) and the cosine of the solar zenith angle (*μ*) on 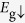 are thoroughly considered, leading to an effective parameterization of 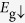 as a nonlinear function of these three quantities. The parameterization is proven able to estimate 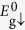 with a mean bias error of 0.32 W m^−2^, which is one order of magnitude smaller than that derived using earlier linear or nonlinear functions. Applications of this new parameterization to estimate *τ*_a_ from 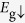, or vice versa, show that the root-mean-square errors were 0.08 and 10.0 Wm^−2^, respectively. Therefore, this study establishes a straightforward method to derive 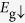 from *τ*_a_ or estimate *τ*_a_ from 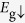 measurements if water vapor measurements are available.

Surface irradiance, the downwelling solar radiation from the Sun and sky that reaches the surface (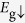), is the ultimate energy source for the Earth’s climate system and life on the planet. A large number of diverse surface processes are governed by the amount of 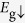; for example, evaporation, snow/glacier melt and plant photosynthesis. Therefore, 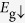 plays an important role in studies of hydrological and carbon cycling^1^,^2^,^3^. Aerosols scatter and absorb solar radiation and thereby modulate the amount of clear-sky 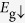 (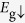 hereafter). We define the aerosol direct radiative effect on 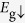 (ADRE) as the attenuation of clear-sky 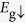 due to aerosol scattering and absorption, i.e., the difference between 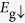 and 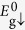 (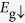 in the absence of aerosols). ADRE is widely reported in the literature, based on a combination of measurements of 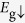 and aerosol optical depth (*τ*_a_)^4^,^5^,^6^,^7^. 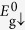 cannot be obtained straightforwardly from observations since the atmosphere almost has aerosols present. One of the difficulties relating to the derivation of ADRE stems from the need to accurately estimate 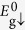
7,8. Radiative transfer model or single-layer clear-sky solar radiation model can be used to calculate 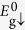
9,10,11,12 and thereby ADRE is obtained, however, this method is sensitive to the calibration uncertainties of pyranometer (independent of model calculation) and dependent on model assumptions about the atmospheric parameters^13^. 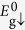 can be estimated from observations, this method avoids dependence on a model, furthermore, ADRE estimation should not be very sensitive to the calibration errors since 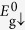 is derived from observations^13^. A linear relationship of 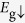 to *τ*_a_ has been popularly assumed and 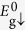 is then derived by linear regression analysis of 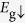 and *τ*_a_^4^,^5^,^13^. Some studies suggested that an exponential decay of 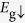 with an increase in *τ*_a_ would be expected according to Beer–Lambert Law, which is especially true for cases with *τ*_a_ values larger than 0.5^7^,^8^,^13^. However, contrary to expectation, a better estimation of 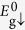 was derived from the linear regression than using the exponential relationship. This was thought to be because the systematic underestimation of 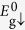 by the linear regression was compensated by the positive correlation between *τ*_a_ and water vapor content (*w*)^8^.

In this study, we show that these previous methods produce a systematic bias in the derivation of 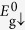 and thereby result in an overestimation of ADRE. A new parameterization of 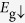 is developed based on global Aerosol Robotic Network (AERONET) data, in which the relationships of 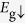 to the cosine of the solar zenith angle (*μ*), *τ*_a_ and *w* have been established by using a combination of nonlinear equations. The results show that the mean bias error (MBE) of the estimations of 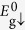 decreases from 4–7 W m^−2^ to 0.32 W m^−2^. The same improvement in the estimation of ADRE would be expected when using the new method. Furthermore, one of the important advantages of this parameterization is that a straightforward method to derive *τ*_a_ from 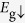, or vice versa, has been established. Specifically, we find it is possible to derive *τ*_a_ from 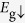 with a root-mean-square error (RMSE) of 0.08, and vice versa with an RMSE of 10.0 W m^−2^.

## Results

**Figure 1:**
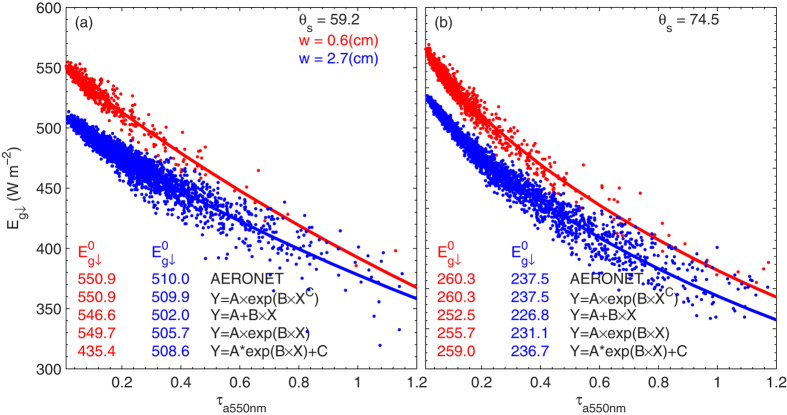
Scatter-plot of aerosol optical depth (*τ*_a_) and surface irradiance (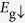) for a solar zenith angle of (a) 59.2° and (b) 74.5°, and water vapor of 0.6 cm (red) and 2.7 cm (blue). These x-marks represent the Aerosol Robotic Network model calculation of surface irradiance in the absence of aerosols (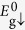). The values given in the first line represent the mean 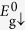 by the Aerosol Robotic Network model calculation, and the following values are 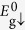 derived on the basis of Eqs (1)–(4). The figure was produced using MATLAB.

**Figure 2:**
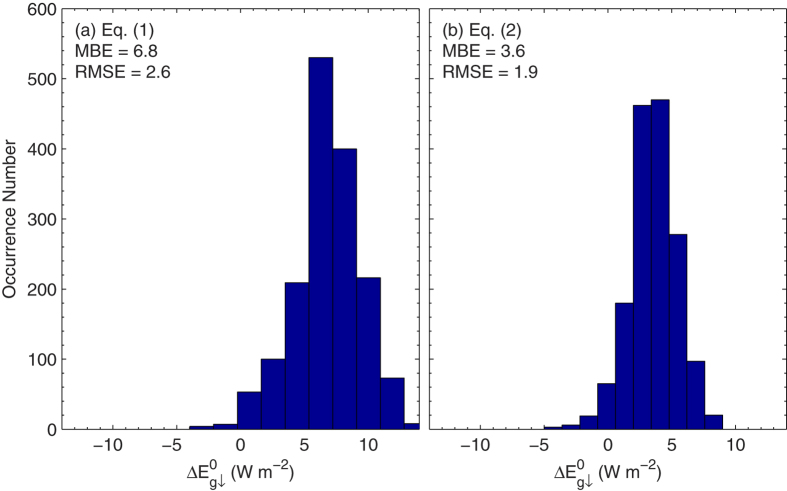
Histogram of the difference in surface irradiance in the absence of aerosols between the Aerosol Robotic Network model calculations and regression results using Eqs (1) and (2) 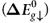. The given values are the mean bias and root-mean-square error of Eqs (1) and (2). The figure was produced using MATLAB.

The solid dots in Fig. 1 represent the scatter between *τ*_a_ at 550 nm (*τ*_a_ hereafter) and 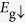 at two narrow *μ* and *w* ranges. The analysis is firstly performed on data points with a very narrow range of *μ* (~0.2°) and *w* (10%*w*), to isolate the effect of *τ*_a_ on 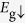. The mean 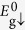 amounts, using the AERONET calculation and fitting 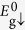 values using regression analysis on the basis of Eqs (1)–(4) in the Method section, are also presented. The performance of these equations is evaluated by the agreement in instantaneous 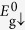 between the AERONET calculations and the regression analysis results. We can see that the best performance is achieved using Eq. (4), the new parameterization proposed in this study, which produces a difference in 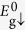 between the mean AERONET calculation and the regression analysis result (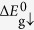) of nearly zero. This nearly zero 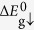 is in fact always derived using Eq. (4) for the full *μ* and *w* ranges (not shown). In contrast, 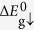, when using Eqs (1)) and (3), varies from a few to tens of W m^−2^ in these two cases. The fact that Eq. (3) occasionally produces unrealistic results that indicate the poor performance of the nonlinear regression analysis^8^, we eliminate it hereafter. Poorer performance of Eqs (1) and (2) than Eq. (4) is further shown by the histogram of 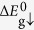 for Eqs (1) and (2) given in Fig. 2. Both equations nearly always underestimate 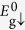. The mean bias error (MBE) and RMSE of 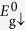 estimations are 6.8 (2.6) W m^−2^ for Eq. (1) and 3.6 (1.9) W m^−2^ for Eq. (2). The fact that Eq. (1) produces a considerably poorer result than Eq. (2) clearly shows the superiority of using nonlinear regression to extrapolate 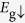 to zero *τ*_a_ rather than linear regression. This conclusion is also supported by the fact that smaller residuals of the regression analysis are derived from Eq. (2) than from Eq. (1). This is expected because attenuation of 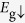 by aerosols shows nonlinear decay, as implied by Beer–Lambert Law.

The difference between Eq. (2) and Eq. (4) is the introduction of a new parameter, *C*, into Eq. (4). The value of *C* is nearly always lower than 1.0 (the exact value of Eq. (3)), which is one of the most important reasons for the better performance of Eq. (4). Eq. (3) is somewhat similar to Beer–Lambert Law, which depicts the attenuation of solar direct radiation by aerosols; however, some part of the attenuation of solar direct radiation is backscattered to the surface, which is certain to enhance 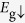. It is therefore expected that *C* should be lower than 1.0. In addition, the effect of aerosols on 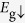 should not be independent from *μ*, since *μ* governs the transfer path of photons. This implies that *C* should vary with *μ*, which is reflected in the following analysis.

**Figure 3:**
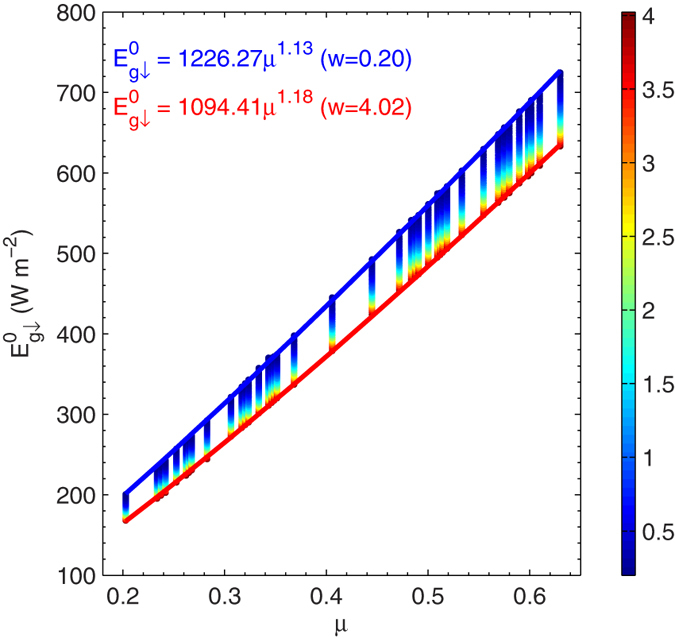
Scatter-plot of the cosine of the solar zenith angle (*μ*) and surface irradiance in the absence of aerosols (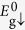). The color bar represents the water vapor content (cm). The curve represents the regression result for a specified water vapor content of 0.20 cm (blue) and 4.02 (red). The figure was produced using MATLAB.

Figure 3 presents the dependence of 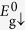 (*A* in Eq. (4)), on *w* and *μ*. Variation of 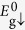 is mainly governed by *μ*, which is somewhat modulated by *w* at the same value of *μ*. Therefore, parameter *A* for a given amount of *w* is firstly simulated using a power law function of *μ* (Eq. (5) in the Methods section). The first and most important reason for the selection of a power law function is because it models the simple physics of the situation with only two parameters^14^,^15^. Parameter *a*_1_ of Eq. (5) represents expected measurements of 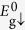 for a *μ* of 1. Parameter *a*_2_ governs the 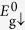 variation with *μ*. The second reason for selection of a power law function is that it provides a faithful approximation to the data.

**Figure 4:**
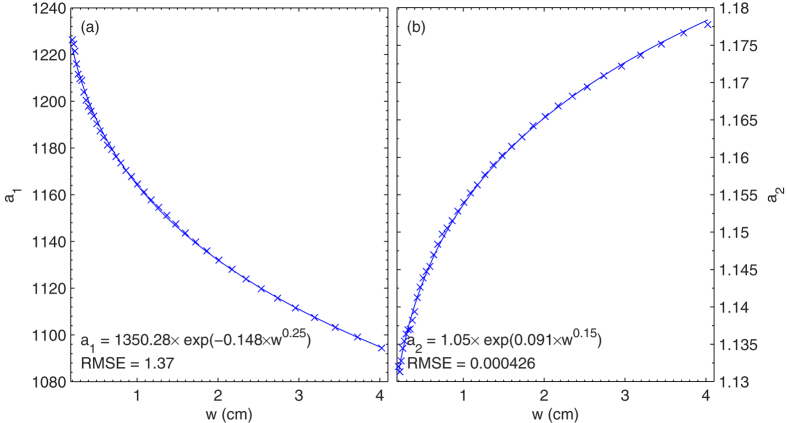
Scatter-plot of water vapor content and parameters (a) *a*_1_ and (b) *a*_2_ ofEq. 5. The curve represents the regression result using Eqs (6) and (7). The figure was produced using MATLAB.

Since the dependence of 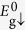 on *μ* is depicted by parameters *a*_1_ and *a*_2_ of Eq. (5), the *w* effect on 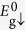 is further simulated through the parameterization of *a*_1_ and *a*_2_ as a function of *w*. Figure 4 shows the relationships of *a*_1_ and *a*_2_ to *w*. We can see that the effect of water vapor per one unit of *w* on 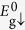 decreases as *w* increases, which is expected since the relative increase in water vapor absorption gradually decreases as *w* increases^16^. These relationships are simulated using an exponential equation. The RMSEs of the regression analysis for *a*_1_ and *a*_2_ of Eq. (5) are 1.37 and 0.0004, respectively, indicating a faithful approximation. A parameterization of 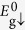 to *μ* and *w* can then be established through a combination of Eqs (5)–(7), 128, 129 that leads to Eq. (8). Therefore, it is straightforward to calculate 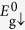 from Eq. (8) if *w* is available, since *μ* can be calculated from location and time very accurately.

**Figure 5:**
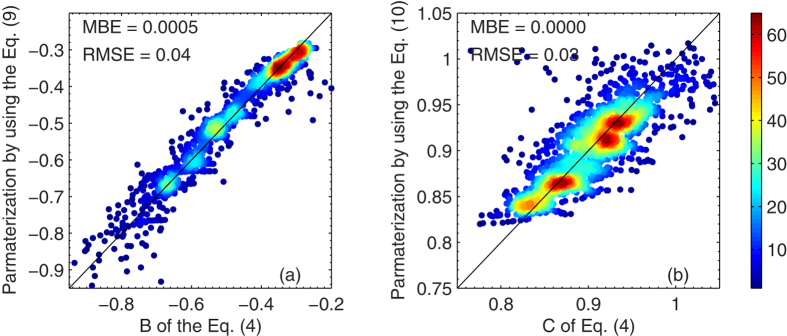
Density plot of parameters (a) B and (b) *C* of Eq. 4 and their parameterization results using Eqs (9) and (10). The color scale represents the relative density of points, where orange to red colors (levels ~45-60) indicate the highest number density. The mean bias error and root-mean-square error of the parameterization are also included. The figure was produced using MATLAB.

Further analysis of parameters *B* and *C* of Eq. (4) shows that both parameters are moderately related to *μ* and *w*, which thereby leads to a parameterization of 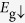 as a function of *τ*_a_, *μ* and *w*. As shown in Fig. 5, parameters *B* and *C* are approximated well using Eqs (9) and (10). In terms of the variability of both parameters, 99.8% is explained by the regression analysis. Since the relationship between instantaneous 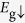 to *τ*_a_ as well as *w* for a specified value of *μ* is established, therefore, a straightforward method is developed that can be used to derive 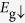 if *τ*_a_ and *w* are available from another source, such as satellite remote sensing. On the other hand, it can also be used to derive *τ*_a_ if 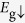 and *w* are available from sources such as the Baseline Surface Radiation Network (BSRN). The proposed method is evaluated by using the 20% of validating data and BSRN data at Xianghe.

**Figure 6:**
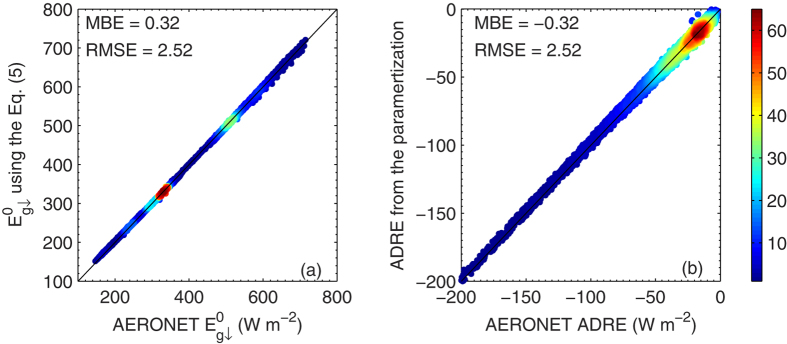
Density plot of AERONET (a) 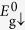 and (b) ADRE and their parameterization results using Eqs (8). The color scale represents the relative density of points, where orange to red colors (levels ~ 45-60) indicate the highest number density. The mean bias error and root-mean-square error of the parameterization are also included. The figure was produced using MATLAB.

Figure 6a shows the comparison of instantaneous AERONET 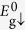 values of validating data and calculations from Eq. (8) based on validating AERONET *τ*_a_ and *w*. The MBE is 0.33 W m^−2^, one order magnitude smaller than the results from Eq. (1) and (2), even though the latter is derived from the training data. Since 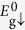 is simulated well by Eq. (5), the ADRE derivation based on Eq. (5) should be very close to that derived from the AERONET model calculations. This expectation is supported by Fig. 6b, in which the AERONET ADREs are compared with estimations from Eq. (8). The MBE and RMSE values are −0.32 and 2.52 W m^−2^, respectively.

**Figure 7:**
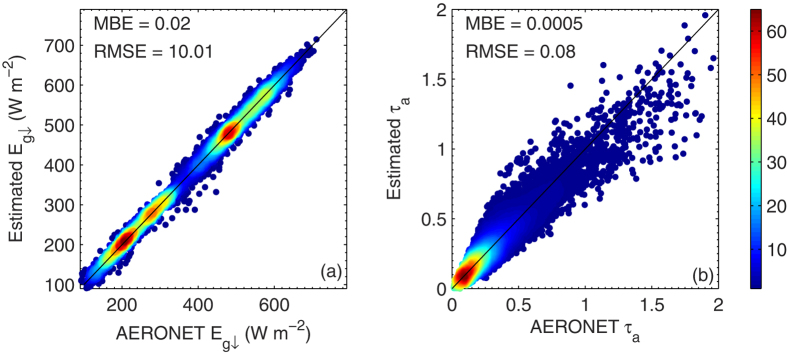
Density plot of AERONET (a) 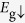 and (b) *τ*_a_ and their parameterization results using Eqs (8-10) . The color scale represents the relative density of points, where orange to red colors (levels ~ 45-60) indicate the highest number density. The mean bias error and root-mean-square error of the parameterization are also included. The figure was produced using MATLAB.

To test the effectiveness of the parameterization of 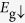, the instantaneous AERONET *τ*_a_ and *w* values from the testing data points are substituted into Eqs (8)–(10), 132, 133 to estimate 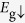 values that are then compared with the AERONET 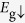 products. Similarly, *τ*_a_ values are estimated from AERONET 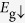 and *w* values and compared with AERONET *τ*_a_ products. Figure 7a shows that 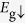 can be estimated with an MBE and RMSE of 0.02 and 10.0 W m^−2^, respectively. This certainly relies on the fact that both *τ*_a_ and *w* are available. On the contrary, if *w* and 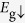 are available and *τ*_a_ is not known, *τ*_a_ can be retrieved from 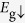 and *w* using this parameterization. The MBE and RMSE values of *τ*_a_ retrievals are 0.0005 and 0.08, respectively (Fig. 7b).

**Figure 8:**
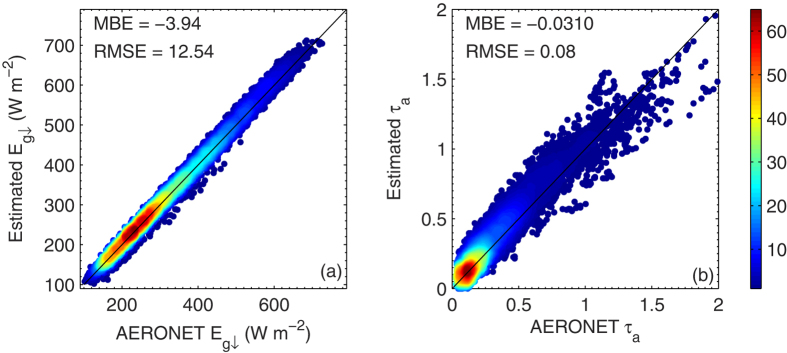
Similar as Fig. 7 but for the results from AERONET and BSRN data at Xianghe. The figure was produced using MATLAB.

Measurements of 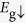 and *τ*_a_ at Xianghe^7^, a BSRN and AERONET station in China are used to further evaluate the effectiveness of the parameterization of 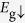. The results are shown in Fig. 8. The estimations of 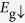 from AERONET *τ*_a_ and *w* products using the proposed parameterization method agree with the BSRN measurements very well, with an MBE and RMSE of −3.9 and 12.5 W m^−2^, respectively. On the other hand, the retrievals of *τ*_a_ from the measurements of 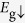 and *w* are compared with AERONET *τ*_a_ products and the MBE and RMSE values are −0.03 and 0.08, respectively. These results once again proved the reliability of the proposed parameterization method.

### Uncertainty analysis

**Figure 9:**
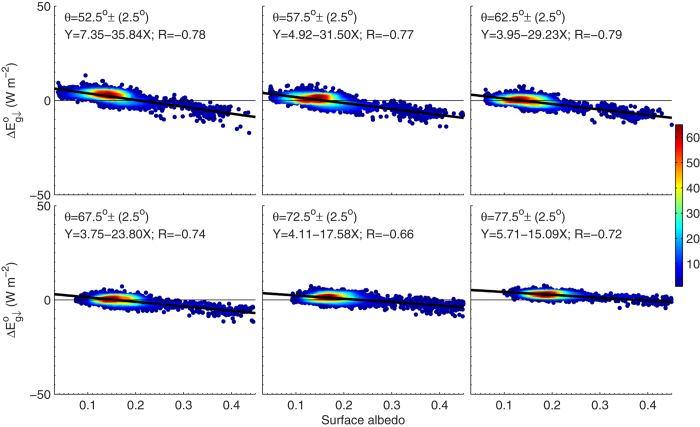
Scatter-plot of shortwave surface albedo to the difference in 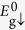 between AERONET product and estimation using the proposed method for six solar zenith angle ranges. The figure was produced using MATLAB.

In the parameterization of 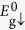 (Eq. 8 of the Method section), surface albedo effect was excluded that likely produced bias in the estimation of 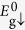. Figure 9 shows the scatter-plot of surface albedo and 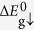, from which we can see a significant negative correlation between both quantities. Uncertainty of 0.1 in surface albedo may produce 1–3 W m^−2^ bias in 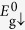.

**Figure 10:**
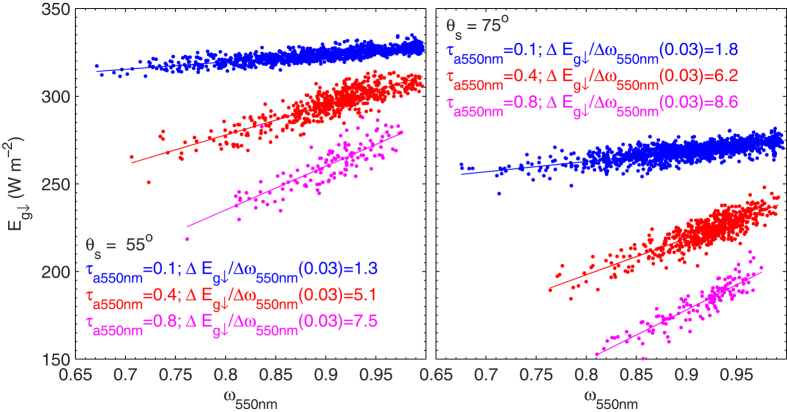
Scatter-plot of *ω*_550*nm*_ to 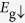 for three *τ*_a_ values and two solar zenith angles. The figure was produced using MATLAB.

**Figure 11:**
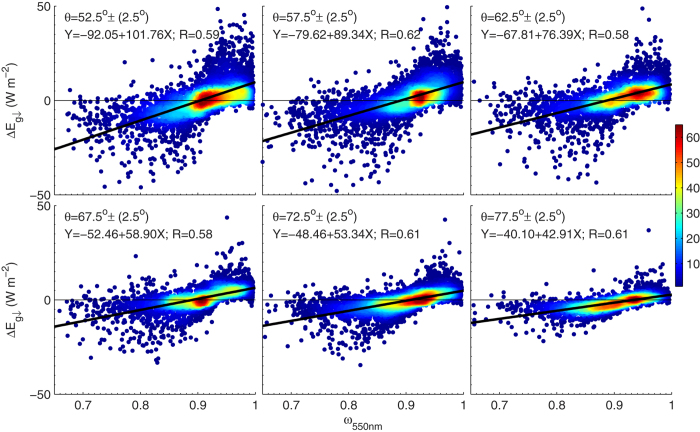
Scatter-plot of *ω*_550*nm*_ to the difference in 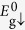 between AERONET product and estimation using the proposed method for six solar zenith angle ranges. The figure was produced using MATLAB.

*τ*_a_ is the dominant aerosol optical property driving the variation of 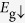 and therefore ADRE. However, aerosol absorption also plays an important role in ADRE^17^, which shows a wide range of variations and thereby may lead to uncertainties in the parameterization of 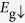, since it was not accounted for. Remarkable impact of aerosol single scattering albedo at 550 nm (*ω*_550nm_) on the estimation of 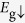 is presented in Fig. 10. 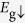 changes as a result of uncertainty of *ω*_550nm_ (0.03) was estimated to be a few Wm^−2^ that depends on optical path and *τ*_a_. Significant impacts of *ω*_550nm_ on estimation of 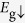 from *τ*_a_ are further evidenced in Fig. 11 in which 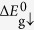 shows a significant correlation to *ω*_550nm_. The best estimation is achieved for *ω*_550nm_ of ~0.90 that is close to the median value of *ω*_550nm_ of AERONET data points.

In the above error analysis of 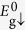, water vapor is assumed to be known without any uncertainty. This is, of course, not realistic, since water vapor products from AERONET, satellite measurements are not free of uncertainty. By differentiating Eq. (8) with respect to *w*, the uncertainty of 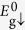 is estimated to be <3 W m^−2^ that is slightly dependent on optical path if the uncertainty of *w* is assumed to be <10% of *w*. The uncertainty of 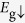 estimation was estimated to <2 W m^−2^ if AERONET *τ*_a_ products with uncertainty of 0.01 ~ 0.02 are used. However, this may reach 10 W m^−2^ if satellite *τ*_a_ products are used since their uncertainty was estimate to be 20% of *τ*_a_ over land^18^. The uncertainly of BSRN 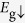 measurements is estimate to be 2%^19^, which may lead to the uncertainty of *τ*_*a*_ < 0.02.

## Discussion


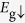
 is one of the key parameters governing a large number of diverse surface processes, and therefore accurate measurement or estimation of 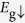 is significant^1^,^2^,^3^. Surface 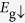 networks are still limited in spatial coverage; therefore, satellite remote sensing is a promising method for the accurate estimation of clear sky 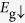. Some highly complex algorithms have been developed to estimate 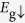 from satellite remote sensing data^20^. Given that *τ*_a_ values have been inverted from a few spaceborne radiometers since 2000 with good quality (e.g. the Moderate Resolution Imaging Spectraradiometer^18^), establishment of the parameterizations in this study provide a straightforward method to calculate clear sky 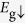 from such satellite aerosol products.

Broadband pyranometer measurements have been used to retrieve *τ*_a_, which is e expected to be a promising method to build a long-term dataset of aerosol loading, since early pyranometer measurements can be tracked to the beginning of the last century^1^. Broadband direct solar radiation is widely used in these previous studies^21^,^22^,^23^. The method proposed in this study is based on global solar radiation measurements that are available more often than direct solar radiation. For example, there are only dozens of stations with direct solar radiation measurements; however, global solar radiation is measured at more than 100 stations in China. Certainly, it should be noted that measurement of global solar radiation is impacted by contamination of the upward facing glass dome, leveling of the instrument and cosine response of the pyranometer. The disadvantage of using direct solar radiation measurements is that it is occasionally disturbed by solar tracker malfunctions. Furthermore, both methods are impacted by calibration uncertainties.

The reason for only considering *τ*_a_ in the proposed method is that the availability of aerosol absorption is very limited, especially from satellite remote sensing. Similar analysis can be performed for a specified area characterized by a special aerosol type, e.g. dust aerosol in desert regions or biomass-burning aerosol in tropical forest regions. In this case, better performance of the parameterization is expected since aerosol absorption shows much less variation for the same aerosol type^24^. Furthermore, lower variation of surface albedo, ozone amount and surface elevation is also expected to reduce the random error of the parameterization.

## Conclusion

Solar zenith angle, aerosol and water vapor are the three most important physical quantities governing the variability of 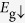. Based on a large quantity of AERONET *τ*_a_, *w*, and 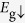 products, the effects of these quantities on 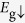 are fully considered, leading to an effective parameterization of 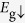 as a nonlinear function of these three quantities. The first advantage is that an accurate estimation of 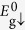 is achieved, which ultimately results in a significant improvement of ADRE estimation compared to previous methods. The second is that a straightforward method has been established to estimate 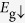 from *τ*_a_, or vice versa, if *w* is available. It is expected that potential applications of this new parameterization in the estimation of 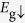 and *τ*_a_ will arise in the near future.

## Methods

I used 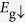, 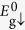, *τ*_a_ and *w* products from those Aerosol Robotic Network (AERONET) sites with an elevation of less than 0.8 km (to eliminate the Rayleigh scattering effect on the analysis) (http://aeronet.gsfc.nasa.gov). The AERONET is a federation of ground-based remote sensing aerosol networks that is composed of more than 700 stations across the world (see Supplementary Fig. S1)^25^. The AERONET products were used because they cover different aerosol types (0 < *τ*_a_ < 3.0; 0.65 < *ω*_550*nm*_ < 1.0; −0.2 < *α*_440_870*nm*_ < 2.5, see Supplementary Fig. S2) and thereby realistically represent the aerosol direct effect on 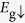. Furthermore, the availability of 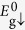 data provides a benchmark for the evaluation of the parameterizations. Uncertainty of *τ*_a_ was estimated to be 0.01–0.02^26^. 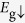 and 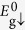 were calculated using the discrete ordinates radiative transfer model with and without aerosols^27^. The 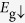 values agree with pyranometer measurements, with the relative difference varying from 0.98 to 1.02^28^,^29^. Of the 950,000 AERONET data points with surface albedo at 440 nm less than 0.25 (to reduce surface albedo effect on the analysis), I randomly select 80% of data points to develop the parameterization and the remaining 20% were used as test data. The analysis flow chart was presented in Supplementary (Fig. S3) that was described as follows.

To isolate the dependence of 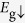 on *τ*_a_, the AERONET data were firstly divided into subgroups according to *θ*_*s*_ and *w*. The range of *θ*_*s*_ was 0.2°. The range of *w* was 10% of *w* (the measurement uncertainty^25^), respectively. The amount of 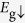 was normalized for the average Earth–Sun distance and cosine correction of 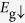 was performed within ranges to its midpoints. Three equations used in the literature were considered to represent the dependence of 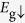 on *τ*_a_:


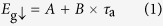



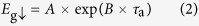



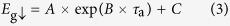


These equations were compared with the following new equation proposed in this paper:


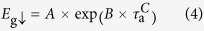


The performance of these methods was evaluated using the 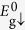 difference between the mean AERONET model calculations and the regression analysis result (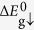). Given that Eq. (3) occasionally produces unrealistic results, we eliminated it in the comparison.

To derive 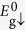 for varying *μ* and *w*, 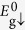 was further parameterized as follows:


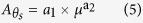


where *a*_1_ and *a*_2_ was found to relate to *w* as follows:


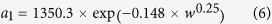



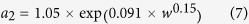


Therefore, the parameterization of 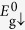 was finally developed through a combination of Eqs (5), (6) and (7).


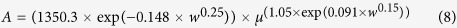


It was found that parameters *B* and *C* of Eq. (4) show moderate variation with *μ* and *w*, which was then simulated by the following equations:


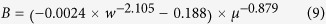



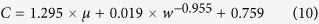


The parameterization of 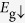 to *μ*, *w* and *τ*_a_ was finally established. This parameterization can be used to estimate 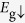 by using a combination of Eq. 4 and 8–10 if *τ*_a_ and *w* are available, and conversely, *τ*_a_ can be directly calculated from 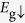 and *w*. *μ* can be accurately calculated from location and time.

## Additional Information

**How to cite this article**: Xia, X. Parameterization of clear-sky surface irradiance and its implications for estimation of aerosol direct radiative effect and aerosol optical depth. *Sci. Rep.* **5**, 14376; doi: 10.1038/srep14376 (2015).

## Supplementary Material

Supplementary Information
