# Supplementary material for: Parameterization of clear-sky surface irradiance and its implications for estimation of aerosol direct radiative effect and aerosol optical depth
Source: Sci Rep. 2015 Sep 23;5:14376. doi: 10.1038/srep14376 (PMC4585779; doi:10.1038/srep14376)
Supplement: Supplementary Information [file srep14376-s1.pdf]

SUPPLEMENTARY INFORMATION

Parameterization of clear-sky surface irradiance and its implications for estimation of aerosol direct radiative effect and aerosol optical depth

Xiangao Xia

LAGEO, Institute of Atmospheric Physics, Chinese Academy of Sciences, Beijing, China

Corresponding Author: Xiangao Xia, LAGEO, Institute of Atmospheric Physics, Chinese Academy of Sciences, Beijing, China. Email: xxa@mail.iap.ac.cn; Tel: 86-10-82995071; Fax: 86-10-82995073.

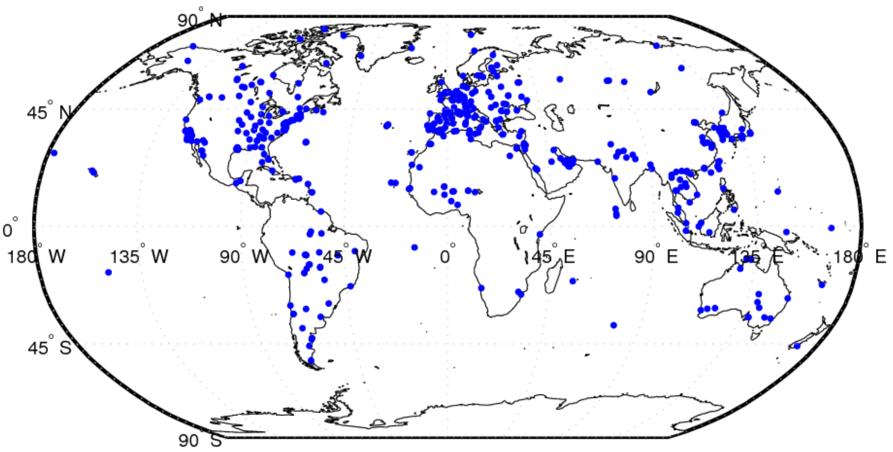

Fig. S1. Spatial distribution of AERONET stations used in the study. The figure was produced using MATLAB.

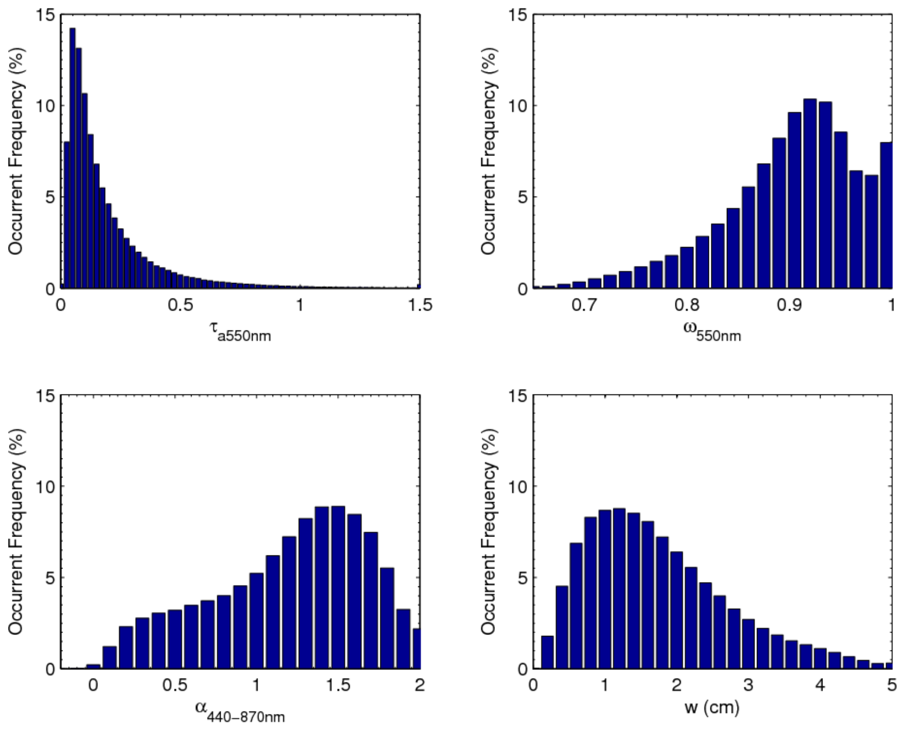

Fig.S2. Histogram of aerosol optical depth at 550 nm, single scattering albedo at 550 nm, Angstrom wavelength exponent and water vapor content. The figure was produced using MATLAB.

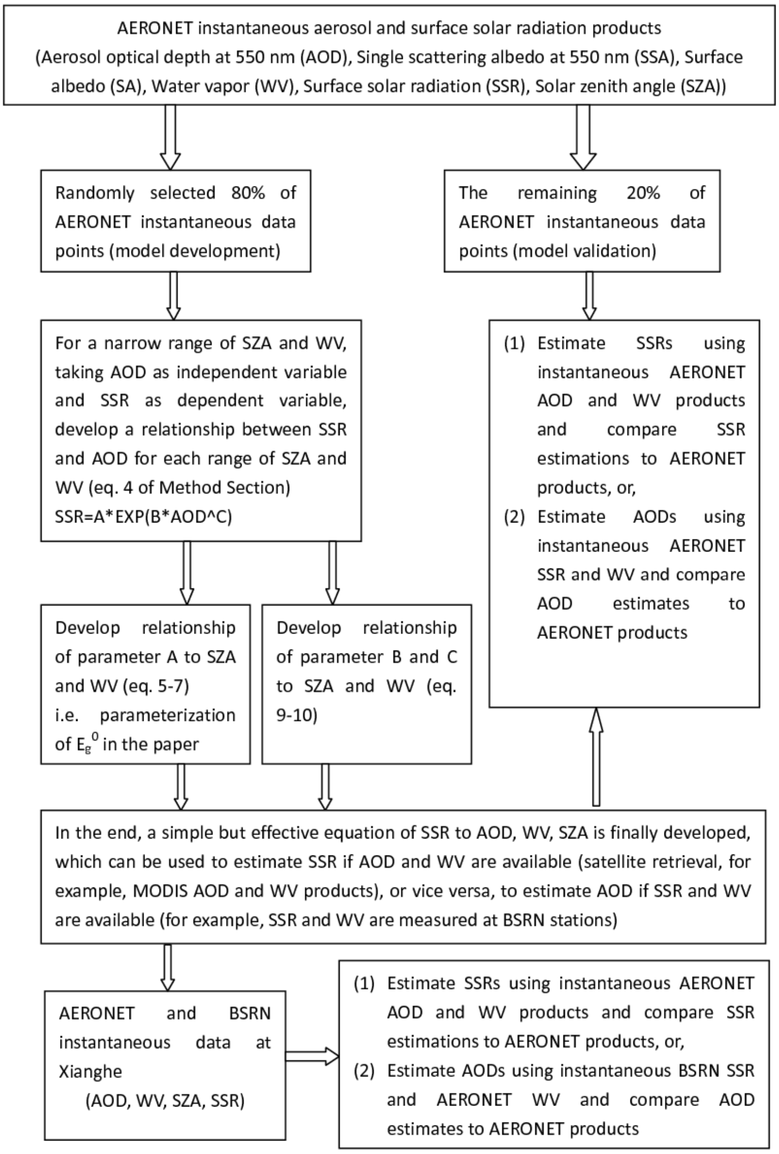

Fig.S3. Flow chart of developing the parameterization of surface solar radiation to aerosol optical depth, water vapor and solar zenith angle using the training data points as well as validating this parameterization using the validating data points. In the establishment of the parameterization, surface solar radiation is taken as the dependent variable. The independent variables include aerosol optical depth, water vapor and solar zenith angle.
